# The E2F3/miR-125a/DKK3 regulatory axis promotes the development and progression of gastric cancer

**DOI:** 10.1186/s12935-019-0930-y

**Published:** 2019-08-13

**Authors:** Yihua Pei, Zhiteng Tang, Minjing Cai, Qin Yao, Bozhen Xie, Xin Zhang

**Affiliations:** 1 Central Laboratory, ZhongShan Hospital XiaMen University, No. 201 Hubin South Road, Xiamen, 361004 Fujian China; 2 Department of Pathology, ZhongShan Hospital XiaMen University, Xiamen, 361004 Fujian China; 3 Department of Center of Clinical Laboratory, ZhongShan Hospital XiaMen University, Xiamen, 361004 Fujian China; 4 Department of Spine Surgery, ZhongShan Hospital XiaMen University, Xiamen, 361004 Fujian China; 5 Department of Rehabilitation, ZhongShan Hospital XiaMen University, Xiamen, 361004 Fujian China

**Keywords:** Gastric cancer (GC), E2F3/miR-125a/DKK3 regulatory axis, Metastasis and progression of tumours

## Abstract

**Background:**

Gastric cancer (GC) is one of the most common malignant tumours with high mortality and metastasis rates. E2F3, miR-125a and DKK3 have been reported to be involved in various cancer types, but their detailed roles in GC have not been fully understood.

**Methods:**

A QRT-PCR assay was used to examine the expression of E2F3, miR-125a and DKK3 in metastatic and nonmetastatic GC tissues. DKK3 plasmids, DKK3 shRNA, miR-125a mimic and miR-125a inhibitor were transfected into BGC823 cells to evaluate the biological functions of DKK3 and miR-125a. A scratch wound healing assay and Transwell assay were utilized to determine the migratory and invasive ability of BGC823 cells transfected with the DKK3 plasmids, DKK3 shRNA, miR-125a mimic and miR-125a inhibitor. Moreover, qRT-PCR and WB analysis were used to analyse the mRNA and protein expression levels of metastasis-related genes after proper transfection. The target relationship between miR-125a and the DKK3 mRNA 3′UTR was determined by a dual luciferase reporter assay, while the interaction between E2F3 and miR-125a was analysed by a ChIP assay.

**Results:**

The clinical data showed that the DKK3 expression level in metastatic GC samples was significantly less than that in nonmetastatic GC samples, whereas the E2F3 and miR-125a expression levels in metastatic GC samples were notably greater than those in nonmetastatic GC samples. Moreover, knockdown of DKK3 and overexpression of miR-125a markedly promoted the migratory and invasive abilities of GC cells. Additionally, the protein and mRNA expression levels of metastasis-related genes, including N-cadherin, Vimentin, MMP2 and MMP9, were markedly decreased in the DKK3 and miR-125a inhibitor groups compared to their control groups and markedly increased in the DKK3 shRNA and miR-125a groups compared with the control group. Finally, a dual luciferase reporter assay and ChIP assay showed that E2F3 binds to the miR-125a promoter and that the DKK3 mRNA 3′UTR is a direct target of miR-125a. Furthermore, analysis of Kaplan–Meier curves also confirmed the regulatory role of E2F3 on miR-125a. Additionally, BGC823 cells transfected with E2F3 plasmids and shRNA downregulated and upregulated the expression of DKK3, respectively.

**Conclusion:**

Our results suggested that E2F3 might play a tumour-promoting role in the metastasis and progression of GC by regulating the miR-125a/DKK3 axis.

## Background

Gastric cancer (GC) is the fourth most common malignancy following lung, prostate, and colorectal cancers worldwide and is the leading cause of cancer death, second only to lung cancer [1]. Although there has been a universal decrease in the incidence and mortality of GC worldwide, it was still reported that up to 2017, there were approximately 990,000 people diagnosed with GC worldwide, of whom approximately 738,000 die from this disease every year [2]. Thus, GC remains a major public health issue that seriously threatens human life. Accumulating evidence has shown that control of the disease mainly depends on early diagnosis and proper treatments [3]. However, most patients with GC are either asymptomatic or have nonspecific symptoms in the early stage; therefore, patients are not treated in the optimal treatment period. In fact, a high proportion of patients with GC are diagnosed at an advanced stage [4]. Although current therapeutic strategies, including surgical resection, chemotherapy and radiation therapy, have rapidly developed, the five‑year survival rate of GC remains rather poor (approximately 5–20%), which might be attributed to disease recurrence as a result of metastasis and drug resistance [5, 6]. Except for genetic factors, previous studies have demonstrated that GC is a complex and multistep disease affected by environmental and lifestyle factors, such as *Helicobacter pylori* (*Hp*) infection, Epstein–Barr virus infection, excessive salt intake, and tobacco use [1, 7]. Additionally, epigenetic regulation has been found to participate in the progression of GC [8]. Therefore, clinical and scientific interests are predominantly focused on the discovery of useful potential biomarkers for non-invasive early detection and new effective therapeutic targets in patients with GC.

MicroRNAs (miRNAs), which are a class of small, endogenous, conserved, non-coding RNAs that are typically 18–24 nucleotides in length, suppress mRNA translation of target genes in a complementary base-pairing manner with the 3′-untranslated region (3′-UTR) of mRNAs, and they are frequently observed in various tumours, including GC, and act as tumour suppressors or oncogenic genes [9, 10]. For example, miR-31 inhibits tumour invasion and metastasis by targeting RhoA in human GC [11]; miR-6852 suppresses cell proliferation and invasion by targeting forkhead box J1 (FOXJ1) in GC [12]; and miR-618 restricts metastasis in GC by downregulating the expression of TGF-β2 [13]. Therefore, miRNAs during tumorigenesis of GC have received increasing attention in recent years [14]. miR-125a has been confirmed to be involved in the development of many cancers, such as breast cancer, hepatocellular cancer [15], colorectal cancer [16], and bladder cancer [17]. Moreover, a large number of studies have revealed that miR-125a also plays an important role in GC [9]. For instance, miR-125a targets STAT3 to regulate GC cell migration and invasion and could be an independent prognostic factor in GC by modulating the proliferation of human GC cells [18, 19]. Nevertheless, the upstream and downstream regulatory mechanisms of miR-125a in GC remain unclear.

E2F3, which is a transcription activator, is crucial to multiple cell processes, including the cell cycle, cell differentiation, DNA damage response, cell death, and cancer development; furthermore, it has been verified that it could serve as an unfavourable prognostic predictor for patients with advanced clinical stages of GC [20]. Additionally, bioinformatics analysis identified that E2F3 can combine with miR-125a. Furthermore, dickkopf-related protein 3 (DKK3) has been identified as a tumour suppressor, and DKK3 is typically used as a cancer biomarker and therapeutic target [21]. In addition, bioinformatics analysis also revealed that miR-125a directly targets the 3′-UTR of DKK3 mRNA. Collectively, in this study, we aimed to investigate the roles of E2F3, miR-125a and DKK3 in the progression of GC and the regulatory mechanisms between E2F3, miR-125a and DKK3.

## Materials and methods

### Tissue samples

Thirty nonmetastatic GC tissues and 30 metastatic GC tissues were collected from patients with GC who underwent surgical resection with no systemic or local treatment before surgery at ZhongShan Hospital, XiaMen University, from January 2017 to December 2017. All patients were confirmed to have GC by experienced pathologists who assessed the pathological stage according to tumour-node-metastasis (TNM) staging of the International Union Against Cancer (UICC)/American Joint Committee on Cancer (AJCC) System (2002). All harvested tissues were correctly labelled, quickly frozen in liquid nitrogen and stored at − 80 °C until use.

All of the patients who donated samples were thoroughly informed about the use of samples, and informed consent was also signed. Furthermore, the present study was approved by the Research Ethics Committee of Xiamen University in accordance with the ethical guidelines of the Declaration of Helsinki.

### Cell lines, cell culture and cell transfection

The GES-1 normal gastric epithelial cell line and human gastric adenocarcinoma cell lines, including AGS, BGC-823, MGC-803, MMKN45 and SGC-7901, were purchased from American Type Culture Collection (ATCC). All these cell lines were maintained in our laboratory and cultured in Dulbecco’s modified Eagle’s medium (DMEM; Gibco, USA) supplemented with 10% foetal bovine serum (FBS; Gibco, USA), 100 U/ml penicillin, and 100 mg/ml streptomycin (Gibco, USA) in a humidified 5% carbon dioxide (CO_2_) incubator at 37 °C. Cells were collected during the logarithmic growth phase for subsequent experiments.

Full-length cDNA sequences of DKK3 were cloned into a pcDNA3.1 vector (Invitrogen, USA) to construct a DKK3-overexpressing plasmid (pcDNA-DKK3). shRNA specifically against DKK3 (sh-DKK3) and a negative control (NC) plasmid, miR-125a mimic and miR-125a inhibitor were synthesized by GenePharma Co., Ltd. (Shanghai, China). All oligonucleotides and plasmids were transfected into the BGC-823 cell line by Lipofectamine 2000 (Promega, USA) in accordance with the manufacturer’s specifications.

### Total RNA extraction, complementary DNA (cDNA) synthesis and quantitative RT-PCR (qRT-PCR)

Total RNA from tissues and cells was isolated using TRIzol reagent (Thermo Fisher Scientific, USA) following the manufacturer’s instructions. After assessing the concentration and purity of the total RNAs, reverse transcription was completed with a cDNA synthesis kit using 1 μg of total RNA. Then, qRT-PCR for the detection of expression of DKK3, miR-125a and E2F3 was conducted using a SYBR-Green PCR Master Mix kit (TAKARA, Japan) with an ABI Prism 7900 Sequence Detection System (Applied Biosystems, USA). The qPCR thermocycling conditions were as follows: 95 °C for 42 s, followed by 40 cycles at 95 °C for 10 s and 60 °C for 35 s. The following primers were used for PCR: 5′-ACA CAG ACA CGA AGG TTG GA-3′ (forward) and 5′-CGT CTC CCA CAG ATG TGA TA-3′ (reverse) for DKK3; 5′-ACA CTC CAG CTG GGT CCC TGA GAC CCT TTA ACC-3′ (forward) and 5′-CTC AAC TGG TGT CGT GGA-3′ (reverse) for miR-125a; 5′-TGA CCC AAT GGT AGG CAC AT-3′ (forward) and 5′-CAT CTA GGA CCA CAC CGA CA-3′ (reverse) for E2F3; 5′-CCT GGA TAC CGC AGC TAG GA-3′ (forward) and 5′-GCG GCG CAA TAC GAA TGC CCC-3′ (reverse) for 18S rRNA; and 5′-CTC GCT TCG GCA GCA CA-3′ (forward) and 5′-AAC GCT TCA CGA ATT TGC GT-3′ (reverse) for U6 snRNA. Triplicate reactions of DKK3, miR-125a and E2F3 were normalized with the housekeeping genes 18S rRNA and U6 snRNA, and the relative expression of the RNAs was calculated by the 2^−ΔΔCt^ method.

In subsequent experiments, to determine the migratory roles of miR-125a and DKK3 in GC metastasis, the expression of E-cadherin, N-cadherin, Vimentin, MMP2 and MMP9 mRNA were analysed by qRT-PCR, as indicated for the abovementioned experimental steps in BGC823 cells transfected with the DKK3-overexpression plasmid, DKK3 shRNA, miR-125a mimic and miR-125a inhibitor. The following primers were used: 5′-TGC AGA AAT TAT TGG GCT CT-3′ (forward) and 5′-GCC CAT TGC AAG TTA CAT AC-3′ (reverse) for E-cadherin; 5′-TGC TAC TTT CCT TGC TTC TG-3′ (forward) and 5′-TCT CTG CCT CTT GAG GTA AC-3′ (reverse) for N-cadherin; 5′-CGC CAG ATG CGT GAA ATG G-3′ (forward) and 5′-ACC AGA GGG AGT GAA TCC AGA-3′ (reverse) for Vimentin; 5′-GCG GCA CCA CTG AG GAC T-3′ (forward) and 5′-TGC GGT CAT CAT CGT AGT TG-3′ (reverse) for MMP2; and 5′-GAA AGC CTA TTT CTG CCA GG-3′ (forward) and 5′-TGC AGG ATG TCA TAG GTC AC-3′ (reverse) for MMP9. 18S rRNA served as the internal control.

### Western blotting (WB) analysis

WB analysis was performed according to a standard method. Briefly, total proteins from cell samples were extracted using radioimmunoprecipitation assay (RIPA) buffer containing the protease inhibitor phenylmethanesulfonyl fluoride (PMSF; Beyotime, China). The concentration of the total proteins was detected by a BCA protein assay kit (Beyotime, China). An equal amount of protein (30 μg) from the different groups was resolved by 8–10% sodium dodecyl sulfate–polyacrylamide gel electrophoresis (SDS-PAGE) at 60 V for 2 h and transferred to polyvinylidene difluoride (PVDF; Millipore, USA) membranes at a constant current of 200 mA. After blocking the PVDF membranes with 5% skim milk at room temperature for 1 h, the PVDF membranes were incubated with primary antibodies against E-cadherin (1:1000 dilution; Abcam, USA), N-cadherin (1:2000 dilution; Abcam, USA), Vimentin (1:1500 dilution; Abcam, USA), MMP2 (1:800 dilution; Abcam, USA), MMP9 (1:800 dilution; Abcam, USA) and GAPDH (1:500 dilution; Boster, China) overnight at 4 °C. Following washing with Tris-buffered saline containing 0.1% Tween-20 (TBST) three times, the membranes were further incubated with the corresponding secondary antibodies (including goat anti-mouse IgG and goat anti-rabbit IgG, 1:12,000 dilution; Boster, China) for 1 h at 37 °C. The membranes were washed with TBST three times and finally visualized using an ECL Western blot detection kit (Amersham, USA). Relative expression levels of each protein were normalized to the endogenous control (GAPDH) using ImageJ 1.8.0 software (National Institutes of Health, USA).

### Scratch wound healing assay

Cells were seeded into 6-well plates at a density of 5 × 10^5^ cells per well. After the cells were cultured for 48 h at 37 °C in an atmosphere containing 5% CO_2_, a sterile 200-µl yellow pipette tip was used to lightly scratch the cells at the centre of the 6-well plate. The wounded monolayers were washed with PBS to remove cell debris, and the cells were cultured in an incubator (at 37 °C with 5% CO_2_). Closure of the wound was observed under an inverted microscope (Olympus, Japan) at 0, 6, 24 and 48 h after scratching, and the distance between the two edges was measured. Ten fields of view were randomly selected, and images were acquired at the indicated timepoints. ImagePro Plus version 5.0 software (Media Cybernetics, Inc., USA) was used to analyse all images.

### Cell migration and invasion assay

Evaluation of cell migration was conducted using a 6.5-mm Transwell insert with a polycarbonate membrane pore size of 8.0 µm (Corning, USA), whereas assessment of cell invasion was conducted using the same Transwell insert, but this Transwell insert was pre-coated with Matrigel (Corning, USA). For the cell migration and invasion assays, in addition to the abovementioned material difference of the Transwell insert, all procedures of these two assays were similar. Briefly, transfected cells were seeded at a density of 3 × 10^5^ cells in the upper chamber of the Transwell insert, while 600 µl complete DMEM containing 10% FBS was added to the lower chamber of the Transwell insert. Cell migration or invasion was allowed to proceed for 48 h at 37 °C, and cells in the upper chamber were carefully removed with a cotton swab. Subsequently, the migrated or invaded cells in the lower chamber were fixed with 4% paraformaldehyde for 20 min, stained with 0.1% crystal violet (Beyotime, China) for 5 min and lightly washed with PBS twice. Eventually, the number of migrated or invaded cells in five random fields of view was counted and photographed with a fluorescence microscope (Olympus, Japan) at 100× magnification.

### Dual luciferase reporter assay

Wild-type (WT) and mutated (with a mutation in the region of the predicted miR-125a binding site) DKK3 3′-untranslated region (UTR) sequences were amplified by PCR and further cloned into a pGL3 control vector (Promega, USA). Additionally, the blank plasmid, miR-125a mimic, miR-125a inhibitor, negative control (NC) and NC inhibitor were synthetized and purchased from Sangon Biotech Co., Ltd. (Shanghai, China). BGC823 cells (1 × 10^4^ cells) were plated in a 96-well plate 1 day before transfection. Next, WT DKK3-3′UTR or mutant DKK3-3′UTR was co-transfected with the miR-215a mimic and miR-125a inhibitor into BGC823 cells using Lipofectamine 2000 according to the manufacturer’s instructions (Invitrogen, USA). At 48 h after co-transfection, firefly and Renilla luciferase activities were determined using a Dual-Glo luciferase assay system (Promega, USA).

### Chromatin immunoprecipitation (ChIP) assay

The possible direct interaction between miR-125a and the E2F3 promoter was then examined by ChIP. BGC823 cells were crosslinked with 1% formaldehyde for 15 min at room temperature, and the reaction was stopped with 0.125 M glycine treatment for 5 min. Subsequently, the crosslinked cells were extensively rinsed twice with 5 mL PBS, and the harvested cells were re-suspended in lysis buffer on ice. Next, a sonicator was used to shear chromatin to an average length of 100–500 bp, which was then diluted tenfold in ChIP dilution buffer and centrifuged at 13,000 rpm/min for 10 min to remove insoluble material. Sheared chromatin was immunoprecipitated with 1 μg of an anti-E2F3 antibody (1:100 dilution; Abcam, USA) or normal mouse IgG (1:150 dilution, as a negative control; Abcam, USA) overnight at 4 °C on a rotating wheel. Immunocomplexes were collected with 40 μl Protein G agarose (Invitrogen, USA) and mixed for 2 h at 4 °C. The beads were washed, and crosslinking was reversed with proteinase K (Invitrogen, USA) in 400 μl elution buffer by incubation for 5–6 h at 65 °C. DNA was purified by phenol/chloroform (Invitrogen, USA) extraction, precipitated overnight at − 20 °C, washed with 70% ethanol and ultimately used as the template for qPCR with specialized primer sets for a ChIP-PCR assay. PCR products were analysed by 3% agarose gel electrophoresis and stained with ethidium bromide (EB).

### Immunohistochemical (IHC) staining

The morphological characteristics of the resected tumour tissues, para-carcinoma tissues and normal tissues were characterized by routine hematoxylin and eosin staining, and their phenotypic patterns were characterized by IHC using monoclonal antibodies against DKK3. Immunohistochemical staining for DKK-3 was performed, as described above. A primary rabbit polyclonal anti-DKK-3 antibody (1:200 dilution; cat. on. bs-2686R; BIOSS) and a secondary antibody mouse anti-rabbit IgM/HRP antibody (1:200 dilution, bs-0369 M-HRP, BIOSS) were used for staining.

### Statistical analysis

The data are presented as the mean ± standard deviation (SD). Statistical analysis was conducted with SPSS 18.0 software (IBM, USA). GraphPad Prism 6.0 software (GraphPad Software, USA) was utilized for plotting the data. Statistical differences between two groups were assessed by a one-tailed Student’s *t*-test, and statistical differences between three groups were assessed by one-way analysis of variance (ANOVA). All experiments were repeated at least three times, and *P* < 0.05 indicated statistical significance.

## Results

### Downregulation of DKK3 expression in human metastatic GC tissues promotes the migration and invasion of GC cells

**Fig. 1:**
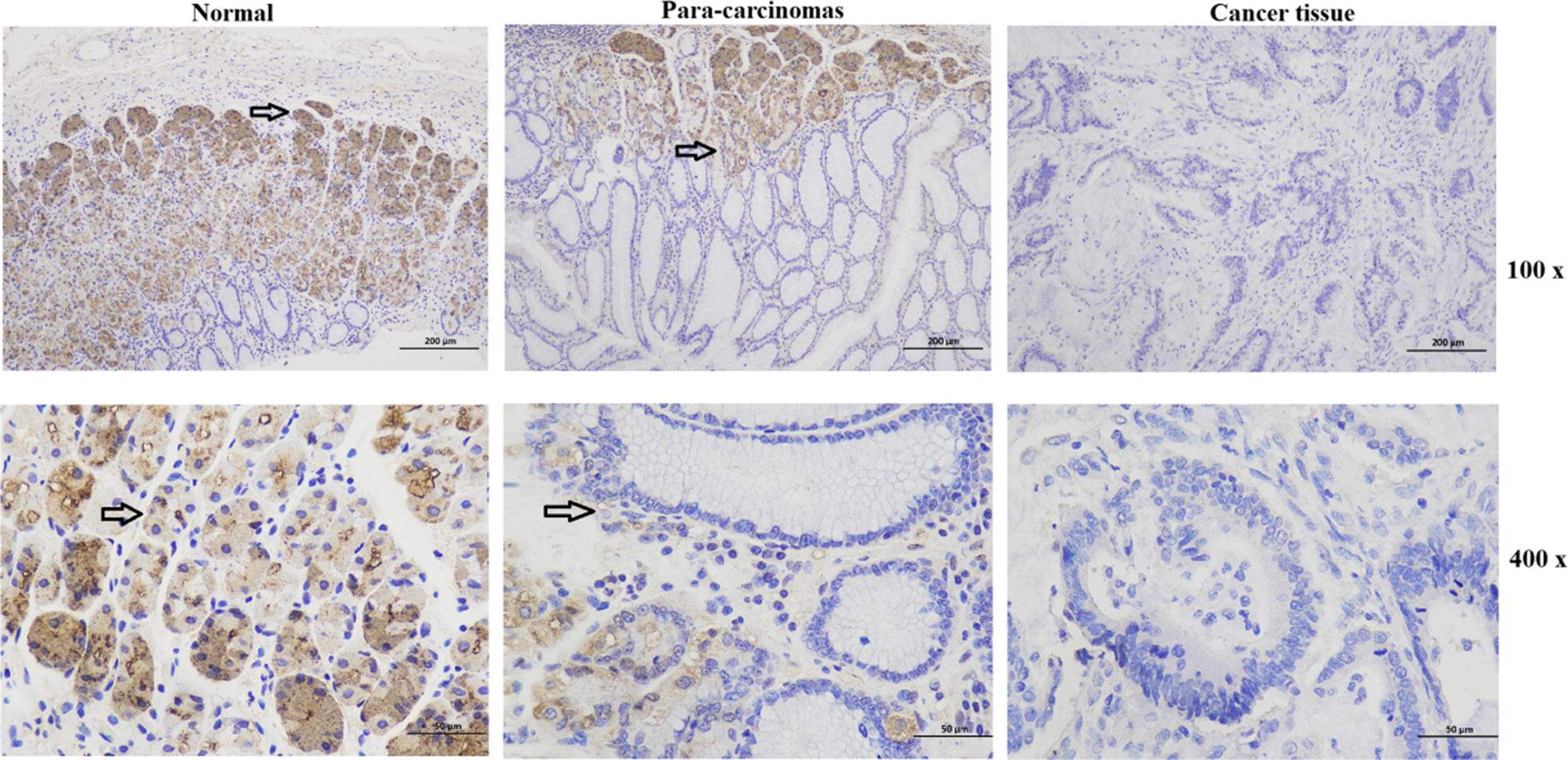
Immunohistochemical staining of DKK3 in the tissues

**Fig. 2:**
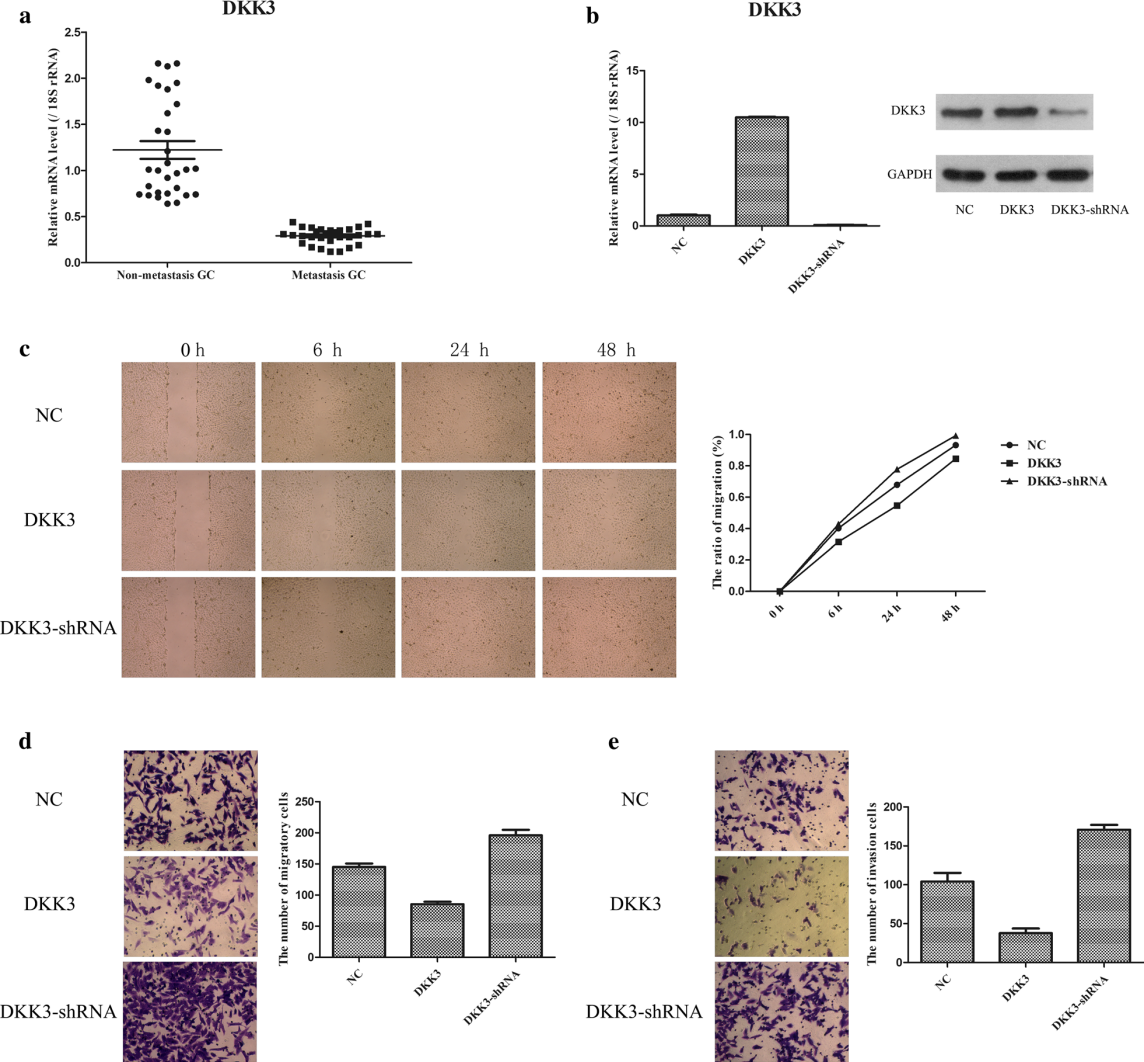
The influences of DDK3 in the development of GC. **a** qPCR was conducted to examine the expression of DDK3 in metastasis-GC samples and non-metastasis-GC samples. **b** The construction of DDK3-overexpression and DDK3-shRNA stably BGC823 cells was performed via transfecting DDK3-overexpression and DDK3-shRNA plasmid. **c** Scratch wound-healing assay was used to measure the migratory ability of GC cells. **d** Transwell assay was utilized to test the migratory ability of GC cells. **e** Transwell assay was applied to detect the invasive ability of GC cells

To investigate the biological role of DKK3 in gastric cancer, we first examined the expression of DKK3 in tissue slices. It was found that DKK3 mRNA was highly expressed in normal tissues, moderately expressed in para-carcinoma tissues and weakly expressed in gastric cancer tissue (Fig. 1). To evaluate the role of DKK3 in the metastasis of GC, we analysed the expression levels of DKK3 in human GC tissues, and the migratory and invasive ability of GC cells was determined after DKK3-overexpression and DKK3 shRNA plasmid transfection. The general and clinical information of 60 patients with metastatic or nonmetastatic GC are shown in Table 1. It was found that metastatic GC samples have a significantly lower level of DKK3 mRNA expression compared with nonmetastatic GC samples (Fig. 2a). Furthermore, the transfected BGC823 cells were analysed by PCR and WB analysis, which showed that both DKK3 mRNA and protein expression levels were markedly increased in DKK3-overexpressing BGC823 cells, while these levels were markedly decreased in DKK3 shRNA-transfected BGC823 cells (Fig. 2b). The role of DKK3 in GC cell metastasis was further examined by a scratch wound healing assay and cell migration/invasion assays. As shown in Fig. 2c–e, the migratory and invasive abilities of BGC823 cells in the DKK3-overexpressing group were noticeably less than those in the NC group, whereas the migratory and invasive abilities of BGC823 cells in the DKK3 shRNA-transfected group were markedly greater than those in the NC group. Therefore, these data suggest that downregulation of DKK3 expression markedly enhances the metastasis of GC tumour cells.

**Table 1:** Demographic and clinical data of patients with non-metastatic and metastatic GC

	Non-metastatic GC (n = 30)	Metastatic GC (n = 30)
Average age (years)	53 ± 7.28	57 ± 6.35
Sex		
Female	10	16
Male	20	14
Clinical staging		
I	9	5
II	11	9
III	8	13
IV	2	3
Grade		
Well-differentiated	15	14
Moderately-differentiated	15	12
Poorly-differentiated	0	4

### Upregulation of miR-125a expression in human metastatic GC tissues promotes the migratory and invasive ability of GC cells

**Fig. 3:**
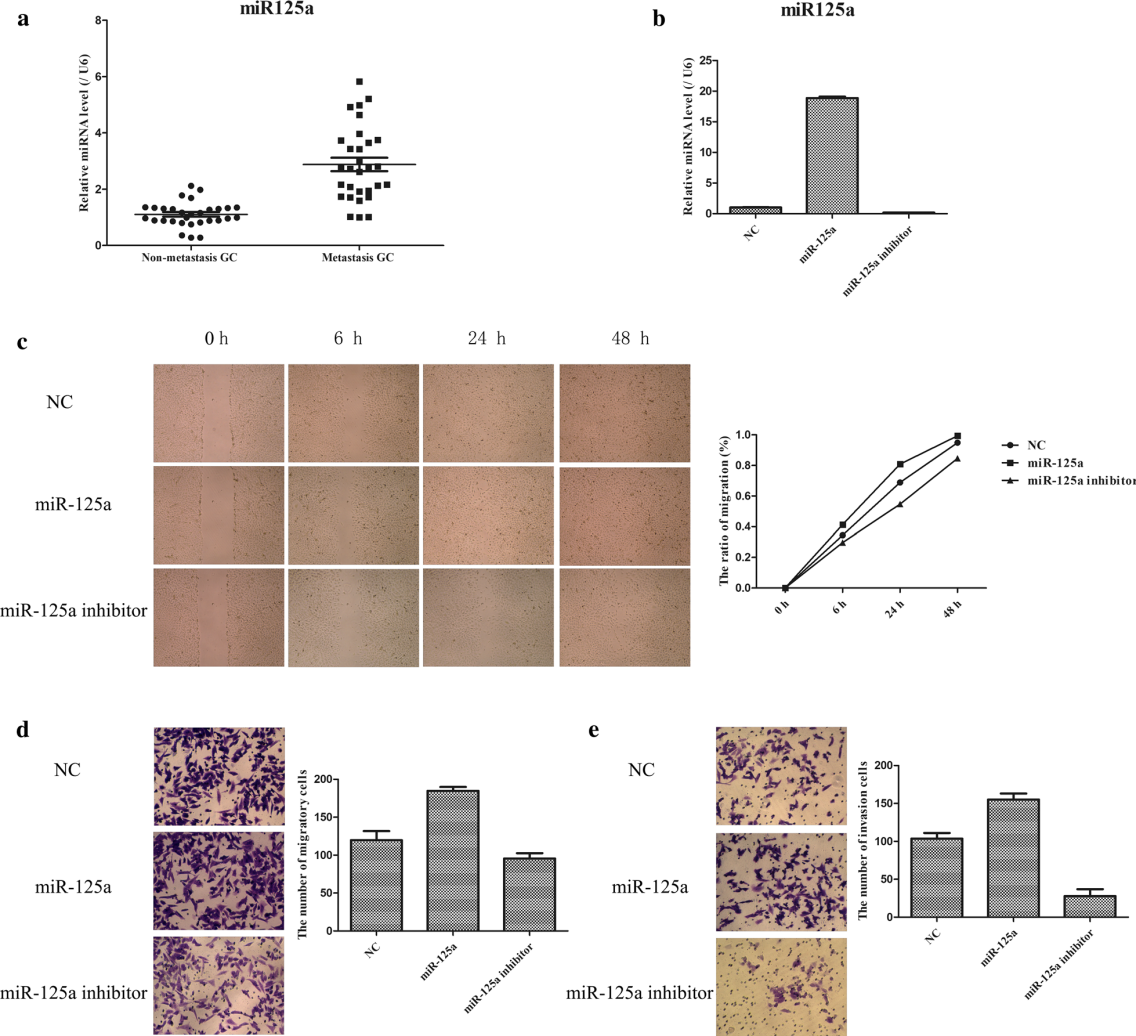
The influences of miR-125a in the development of GC. **a** qPCR was carried out to examine the expression of miR-125a in metastasis-GC samples and non-metastasis-GC samples. **b** The establishment of miR-125a mimic and miR-125a inhibitor stably BGC823 cells was performed via treating with miR-125a mimic and miR-125a inhibitor. **c** Scratch wound-healing assay was used to measure the migratory ability of GC cells. **d** Transwell assay was utilized to test the migratory ability of GC cells. **e** Transwell assay was applied to detect the invasive ability of GC cells

To assess the effects of miR-125a on the metastasis of GC, we analysed the expression levels of miR-125a in human GC tissues. Then, changes in the migratory and invasive abilities of GC cells were analysed after miR-125a mimic and miR-125a inhibitor transfection. It was discovered that the expression level of miR-125a in metastatic GC tissues was greater than that in nonmetastatic GC samples (Fig. 3a). Subsequently, the miR-125a mimic and miR-125a inhibitor were transfected into BGC823 cells to further monitor the role of miR-125a in the migratory and invasive abilities of GC cells. The results showed that miR-125a expression was notably upregulated in miR-125a mimic-transfected BGC823 cells, but it was obviously downregulated in miR-125a inhibitor-transfected BGC823 cells (Fig. 3b). Furthermore, as shown in Fig. 3c–e, cell migration and invasion were markedly enhanced in the miR-125a-overexpressing group compared to the NC group, while cell migration and invasion were clearly suppressed in the miR-125 inhibitor group compared with the NC group. Thus, these results indicated that forced miR-125a expression distinctly accelerated the metastasis of GC tumour cells.

### DKK3 is a direct target of miR-125a

**Fig. 4:**
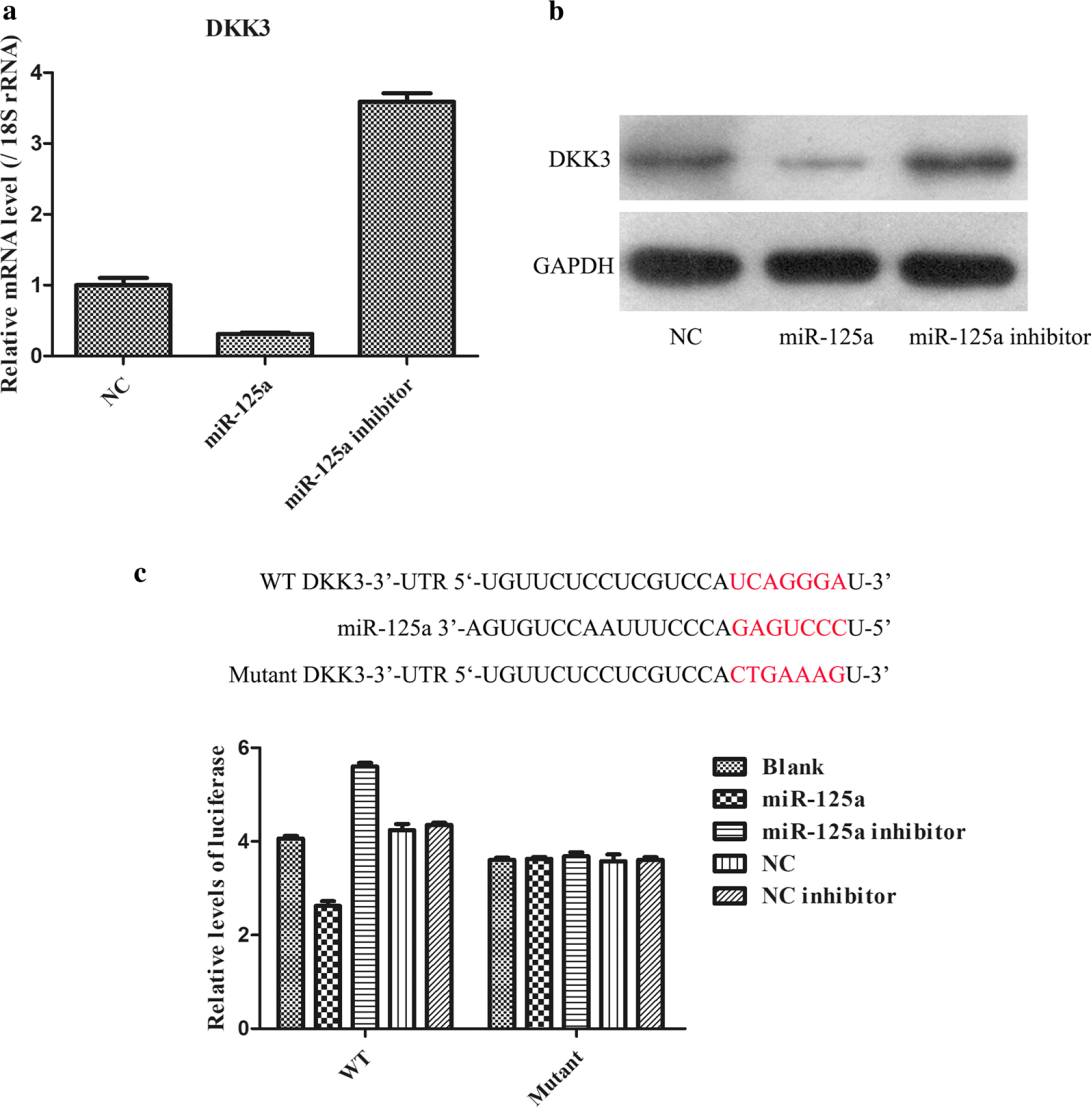
DDK3 is predicted to be a target gene of miR-125a. **a** qPCR analysis to determine the transfected efficiency in changes of DDK3 mRNA. **b** WB analysis to detect the transfected efficiency in changes of DDK3 protein. **c** Dual-luciferase reporter gene assay was performed to indicate the target interaction between miR-125a and DDK3

The abovementioned data revealed a negative linear correlation between the expression of miR-125a and DKK3 in metastatic GC samples, and we speculated that there might be a targeted interaction between miR-125a and DKK3. First, qRT-PCR and WB analysis indicated that the mRNA and protein expression of DKK3 was markedly suppressed in miR-125a mimic-transfected BGC823 cells, while it was significantly elevated by miR-125a inhibitor transfection in BGC823 cells (Fig. 4a, b). Finally, the results from the luciferase reporter assay showed that miR-125a led to a marked reduction in luciferase activity in the DKK3-WT group compared with the blank group but had no obvious effect on the luciferase activity in the DKK3-mutant group (Fig. 4c). Therefore, these findings implied that DKK3 might be a direct target of miR-125a.

### miR-125a regulates GC cell metastasis through targeting DKK3

**Fig. 5:**
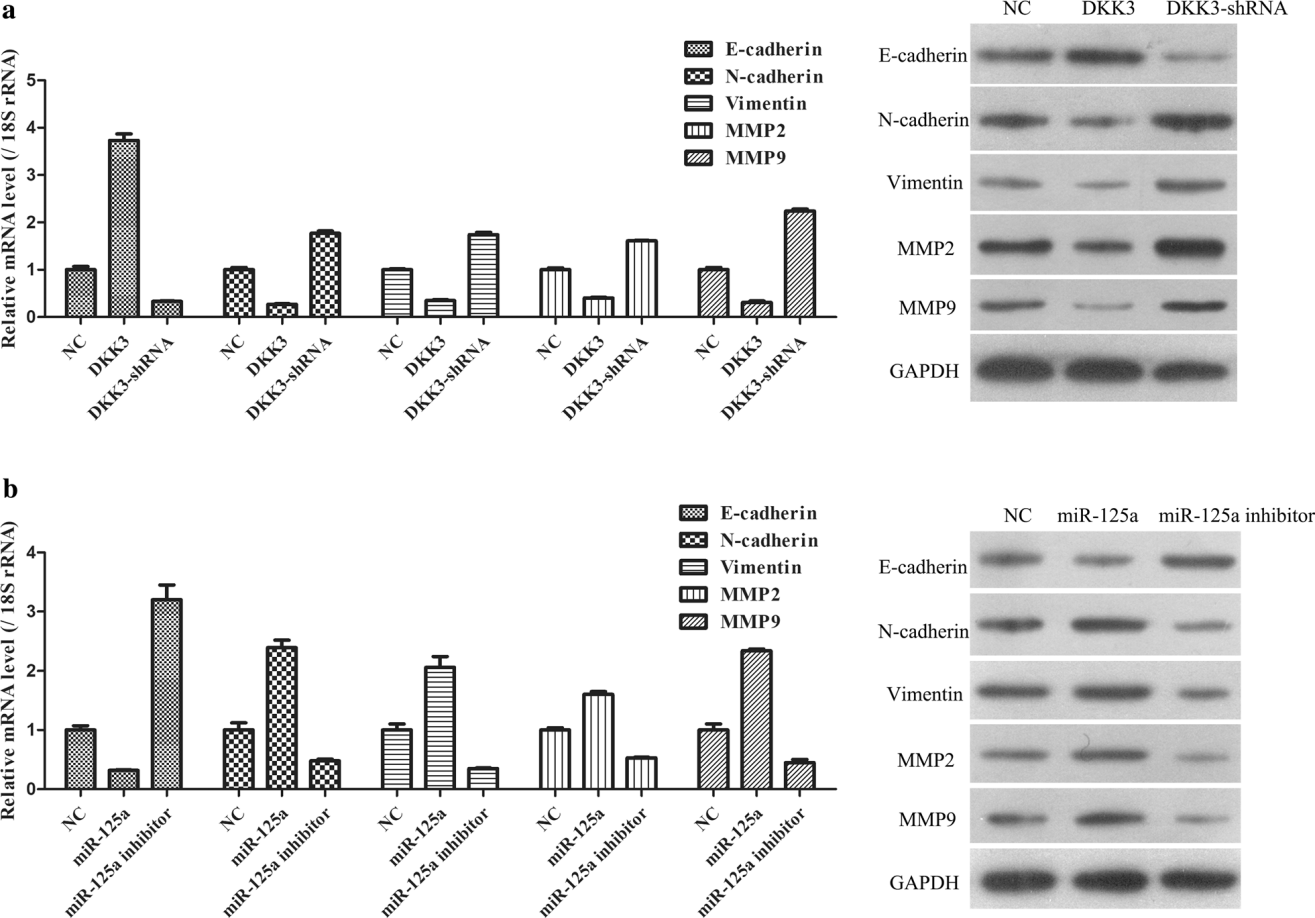
DDK3 and miR-125a trigger similar repressive effects in expressions of metastasis-related genes. **a** qPCR and WB analysis were carried out to examine the mRNA and protein expression levels of E-cadherin, N-cadherin, Vimentin, MMP2 and MMP9 in DDK3-overexpression and DDK3-shRNA treated cells. **b** qPCR and WB analysis were carried out to examine the mRNA and protein expression levels of E-cadherin, N-cadherin, Vimentin, MMP2 and MMP9 in miR-125a mimic and miR-125a inhibitor treated cells

According to the abovementioned study, DKK3 and miR-125a are both closely associated with GC cell metastasis. Furthermore, miR-125a negatively regulates the expression of DKK3 in GC cells. Therefore, it was necessary to further explore the regulatory effect of miR-125a and DKK3 on the expression of cell metastasis-related genes, including E-cadherin, N-cadherin, Vimentin, MMP2 and MMP9. Except for E-cadherin, the mRNA and protein expression levels of the other metastasis-related genes markedly decreased in the DKK3 group compared to the NC group and markedly increased in the DKK3 shRNA group compared to the NC group (Fig. 5a). Furthermore, compared with the DKK3 and DKK3 shRNA groups, the mRNA and protein expression changes of these genes were inversed in miR-125a- and miR-125a inhibitor-transfected GC cells (Fig. 5b). Similar results were observed in the DKK3 group when there was a low expression level of miR-125a in GC cells. Additionally, similar results were observed in the DKK3 shRNA group when there was a high expression level of miR-125a in GC cells. These results further revealed that miR-125a might affect GC cell metastasis by regulating DKK3 expression.

### E2F3 binds to the promoter of miR-125a and regulates miR-125a expression

**Fig. 6:**
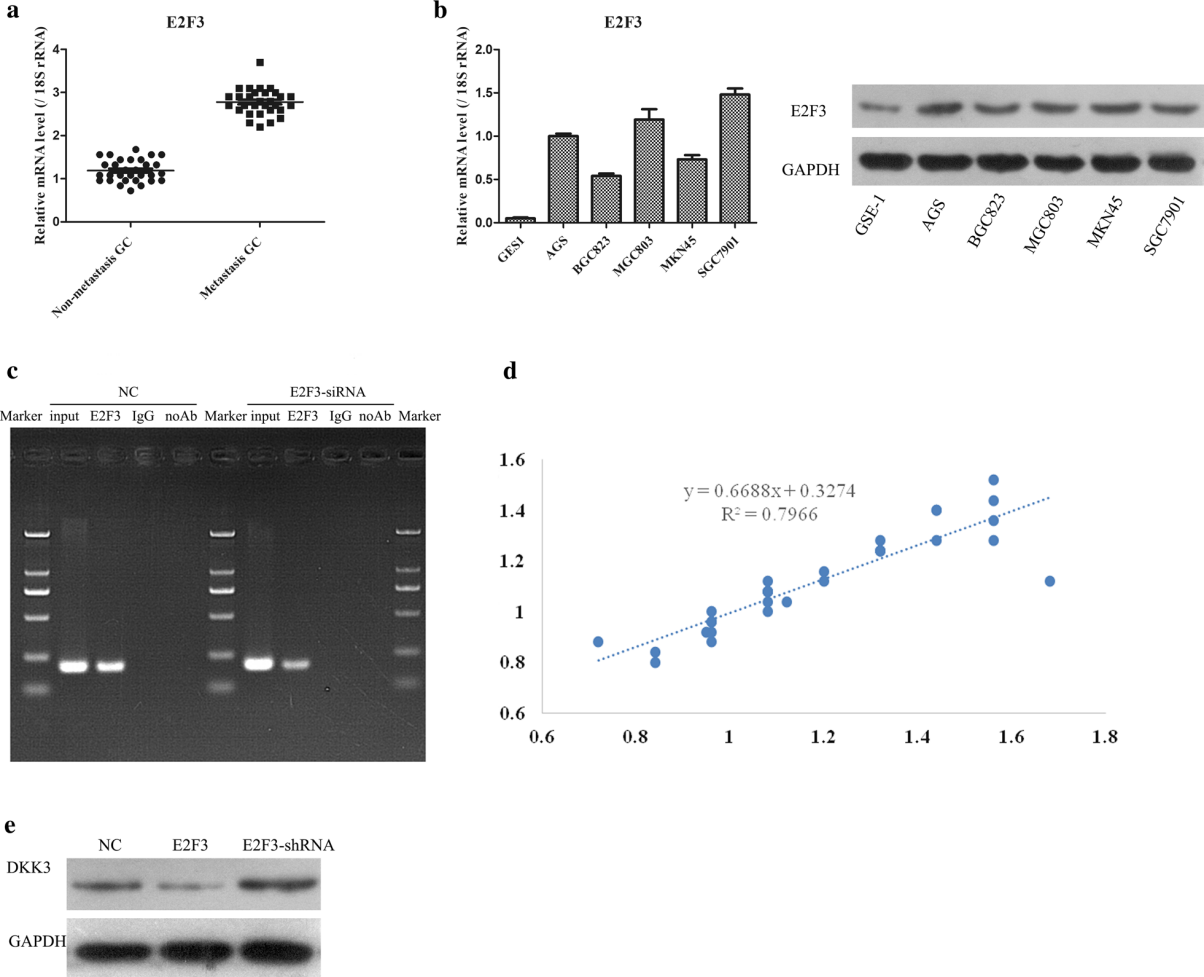
The role of E2F3 in development of GC. **a** qPCR was conducted to examine the expression of E2F3 in metastasis-GC samples and non-metastasis-GC samples. **b** qPCR was performed to test the expression of E2F3 in GC cell lines. **c** ChIP with anti-E2F3 followed by PCR with miR-125a promoter primers in BGC823 cells. **d** Kaplan–Meier curves were constructed to demonstrate the correlation between E2F3 and miR-125a. **e** The effects of E2F3 on the expression of DDK3 were examined by WB

First, the expression of E2F3, which is a transcription factor, was analysed in patients with GC and in cell lines. It was shown that the expression level of E2F3 was significantly increased in metastatic GC samples compared with nonmetastatic GC samples (Fig. 6a). Second, the mRNA and protein expression levels of E2F3 were analysed in GC cell lines by qRT-PCR and WB assays. The data showed that E2F3 expression in the normal gastric epithelial cell line GES-1 was notably less than that in the human gastric adenocarcinoma cell lines (Fig. 6b). To confirm the regulatory role of E2F3, the interaction between E2F3 and miR-125a was identified by a ChIP assay. As shown in Fig. 6c, suppressed expression of E2F3 obviously inhibited miR-125a expression. Moreover, the correlation between E2F3 and miR-125a expression in clinical samples was further evaluated by analysis of Kaplan–Meier curves (Fig. 6d). To demonstrate the regulatory mechanism of E2F3 on DKK3, an E2F3 mimic and E2F3 shRNA were transfected into GC cells, and the results showed that overexpression of E2F3 augmented DKK3 expression, but knockdown of E2F3 weakened DKK3 expression (Fig. 6e). Collectively, these results suggest that E2F3 might be an upstream regulator that dominates the downstream miR-125a/DKK3 axis in GC progression.

## Discussion

For the past few decades, GC incidence and mortality have markedly decreased in most areas of the world, but it is still a disease that is associated with a poor prognosis and outcome, mainly due to its progression and widespread metastasis [8]. Moreover, based on the complex and multifactorial pathogenesis of GC, current treatment methods, such as surgical resection, radiotherapy, chemotherapy, and chemotherapy integrated with Chinese medicine, do not distinctly improve the survival of patients with GC [22, 23]. Thus, a better understanding of the molecular mechanism underlying tumour progression and metastasis may contribute to the development of novel targeted therapies for GC. Studies have demonstrated that abnormalities in genetic factor alterations are closely associated with the occurrence and progression of GC; therefore, investigators have been focused on the genetic aspects of GC to obtain new diagnostic and prognostic markers during tumorigenesis [24]. In this study, the expression levels of DKK3 and miR-125a were decreased and increased, respectively, in metastatic GC samples compared with nonmetastatic GC samples. In fact, increasing evidence has shown that the expression level of DKK3 is decreased in several human solid cancers, including prostate, colon, and breast cancers [21, 25]. Additionally, it has also been reported that miR-125a is a prognostic indicator in patients with GC based on its lower expression level in GC tissues and cell lines [9, 14]. Thus, our data were completely consistent with data from previous studies. Then, the metastatic functions of DKK3 and miR-125a were further explored by a scratch wound healing assay and Transwell assay. These results revealed that a lower DKK3 expression level enhanced the migratory and invasive abilities of GC cells, whereas a lower miR-125a expression level suppressed the migratory and invasive abilities of GC cells. Metastasis is closely associated with a poor prognosis of GC [2, 8]. Nevertheless, migration and invasion are both important events during the process of tumour metastasis [26]. Therefore, we hypothesized that the integrated activity of DKK3 and miR-125a participates in the modulation of GC cell metastasis. Furthermore, according to their opposite expression patterns, we hypothesized that there might be a targeted regulatory interaction between DKK3 and miR-125a. The dual luciferase reporter assay verified our assumption.

For most patients with advanced GC, a poor prognosis is predominantly attributed to tumour metastasis [3]. Furthermore, not only for GC but also for most cancers, tumour metastasis is a substantial challenge for treating cancer [26]. Therefore, we evaluated the expression of many metastasis-related genes in GC cells treated with DKK3 and miR-125a. First, the cadherin superfamily, which is a class of homophilic adhesion molecules with important functions in cell–cell adhesion, tissue morphogenesis, and cancer, encompasses more than 100 members in humans, including classic cadherins, numerous proto-cadherins and cadherin‑related proteins [27, 28]. Numerous studies have indicated that E-cadherin and N-cadherin are key components of cell–cell junctions in epithelial monolayers and are implicated in the growth and invasion of tumours [28]. Second, vimentin, which is a major constituent of the intermediate filament family of proteins that maintains cellular integrity and provides resistance against stress, has been shown to accelerate tumour growth and invasion [29]. Third, MMPs, especially MMP2 and MMP9, that degrade constituents of the extracellular matrix to disrupt the physiological barrier were found to participate in tumour metastasis [30]. Thus, E-cadherin, N-cadherin, vimentin, MMP2 and MMP9 were identified as attractive cancer targets that all play important roles in the context of tumour metastasis. Furthermore, our results showed that, except for E-cadherin, the expression levels of N-cadherin, Vimentin, MMP2 and MMP9 were consistently decreased in the DKK3 and miR-125a inhibitor groups compared to their corresponding control groups and were markedly increased in the DKK3 shRNA and miR-125a groups compared to their corresponding control groups. These results further confirmed that DKK3 and miR-125a alter the tumour metastasis process by regulating metastasis-related molecules.

Ultimately, overexpression of E2F3, which is an oncogene in tumorigenesis, is associated with a poor prognosis in a variety of human malignancies, such as hepatocellular carcinoma [31]. A study of 976 patients with GC indicated that a high expression level of E2F3 is correlated with a poor prognosis compared with a low expression level of E2F3 [20]. Disruptive expression of DKK3 in cancers has been verified in some cancer types, including pancreatic cancer, renal cell carcinoma, thyroid cancer, breast cancer, and colorectal cancer, and downregulated expression of DKK3 has been observed [32–34]. However, in other types of cancer, such as gastric cancer, breast cancer and ovarian cancer, upregulated DKK3 mRNA expression has been found. In GC, patients with a high expression level of DKK3 have a lower overall survival (OS) rate than those with a low expression level of DKK3 [35]. In our study, it was shown that the expression level of E2F3 was markedly increased in metastatic GC samples and GC cell lines, indicating the tumour activator role of E2F3. Additionally, through a more in-depth understanding of E2F3, it was revealed that there are intricate networks between E2F3 and miRNAs in regulating the occurrence, development and progression of tumours [36, 37]. Thus, the relationship between E2F3 and miR-125a was examined by a ChIP assay and analysis of Kaplan–Meier curves. In addition, the regulatory effects of E2F3 on the downstream gene DKK3 of miR-125a were also determined by WB. The result precisely indicated that E2F3 mediates DKK3 by targeting miR-125a.

## Conclusion

Overall, the present study revealed that the expression of DKK3 was frequently downregulated in metastatic GC compared with nonmetastatic GC, but the expression of miR-125a and E2F2 was typically upregulated in metastatic GC. Changes in DKK3 and miR-125a expression were closely associated with tumour metastasis in the progression of GC. Furthermore, E2F3, which is an upstream regulatory molecule, directly targets the miR-125a promoter and further mediates the miR-125a-targeted gene DKK3. Therefore, our findings indicated that E2F3, miR-125a and DKK3 might compose a linear regulatory axis and might be novel targets for GC therapy.

## Data Availability

The datasets during the current study available from the corresponding author on reasonable request.

## Abbreviations

GC: gastric cancer
Hp: *Helicobacter pylori*
miRNAs: microRNAs
3′-UTR: 3′-untranslated region
FOXJ1: forkhead box J1
DKK3: dickkopf-related protein 3
TNM: tumour-node-metastasis
IUCC: International Union Against Cancer
AJCC: American Joint Committee on Cancer
ATCC: American Type Culture Collection
FBS: foetal bovine serum
WB: western blotting
WT: wild-type
ChIP: chromatin immunoprecipitation
IHC: immunohistochemical
